# Selenium Induces Pancreatic Cancer Cell Death Alone and in Combination with Gemcitabine

**DOI:** 10.3390/biomedicines10010149

**Published:** 2022-01-11

**Authors:** David J. Wooten, Indu Sinha, Raghu Sinha

**Affiliations:** 1 Department of Physics, Penn State University, University Park, PA 16802, USA; dzw347@psu.edu; 2 Department of Biochemistry and Molecular Biology, Penn State College of Medicine, Hershey, PA 17033, USA; isinha@pennstatehealth.psu.edu

**Keywords:** selenium, pancreatic cancer, combination, synergy

## Abstract

Survival rate for pancreatic cancer remains poor and newer treatments are urgently required. Selenium, an essential trace element, offers protection against several cancer types and has not been explored much against pancreatic cancer specifically in combination with known chemotherapeutic agents. The present study was designed to investigate selenium and Gemcitabine at varying doses alone and in combination in established pancreatic cancer cell lines growing in 2D as well as 3D platforms. Comparison of multi-dimensional synergy of combinations’ (MuSyc) model and highest single agent (HSA) model provided quantitative insights into how much better the combination performed than either compound tested alone in a 2D versus 3D growth of pancreatic cancer cell lines. The outcomes of the study further showed promise in combining selenium and Gemcitabine when evaluated for apoptosis, proliferation, and ENT1 protein expression, specifically in BxPC-3 pancreatic cancer cells in vitro.

## 1. Introduction

Pancreatic cancer is a highly lethal malignancy and the seventh leading cause of cancer-related deaths in men and women worldwide, according to the 2018 global cancer statistics [1]. The first line of systemic chemotherapy for this cancer type includes Gemcitabine as a single agent [2] or this could be used in combination with other drugs such as nanoparticle albumin-bound (nab)-paclitaxel [3], capecitabine [4,5], or erlotinib [6]. However, these combination therapies can be associated with significant toxicities including peripheral neuropathy (nab-paclitaxel), hand–foot syndrome (capecitabine), and severe diarrhea or severe skin rash (erlotinib). On the other hand, the combination of 5-fluorouracil, leucovorin, oxaliplatin, and irinotecan (FOLFIRINOX) is a commonly used first-line systemic treatment; however, the associated toxicities can be devastating, limiting its use [7]. There is a need to find better combination therapies in pancreatic cancer that may offer better efficacy at lower doses and less associated toxicity.

Selenium is an essential trace element and an important micronutrient required for human health. Selenium is useful in the prevention and/or treatment of various disorders including but not limited to hypothyroidism [8], cardiovascular disease [9], arthropathies (including Kashin–Beck disease, rheumatoid arthritis, osteoarthritis, and osteoporosis) [10], atherosclerosis [11], HIV and AIDS [12], Alzheimer disease [13], and possibly stroke [14] and COVID-19 [15]. Numerous selenium forms have extensively been investigated for their potent anti-tumor activity by inhibiting cell proliferation of several cancer types including but not limited to breast, prostate, lung, melanoma, and cervical [16,17,18,19,20,21,22,23]. However, there are mixed reports on levels of selenium in blood with incidence of pancreatic cancer. Higher levels of selenium in blood might be associated with longer survival in patients with pancreatic cancer [24,25]. Additionally, higher levels of selenium appeared protective for both mutated and KRAS wild-type pancreatic ductal adenocarcinoma [26]. However, there was a weak association between serum selenium and pancreatic cancer in the PLCO cohort [27]. However, in vitro there are limited reports on the use of inorganic selenium [28] and synthetic selenium [29] compounds in combination with Gemcitabine to enhance growth inhibition of pancreatic cancer cells.

In order to measure and indicate beneficial outcomes of combination therapies, appropriate quantitation tools are necessary. The interaction of drugs in a combination is commonly quantified using a synergy reference model [30,31]. A recently developed synergy model, multi-dimensional synergy of combinations (MuSyC), distinguishes distinct types of synergy, allowing for prioritization of, e.g., synergistic efficacy or synergistic potency [32,33]. The MuSyC model has many parameters, which, however, may not be well-constrained by available data. In cases like this, older, nonparametric synergy models such as highest single agent (HSA) [34] are useful to provide quantitative insights into how much better the combination performs than either drug alone.

The current study was designed to study the potential of combining Selenium with Gemcitabine on pancreatic adenocarcinoma in a conventional 2D cell culture (on plastic). Additionally, in order to achieve an effective pre-clinical dose range for a combination of these drugs, we also evaluated the impact of the same in a 3D culture platform. We compared the synergy of Selenium and Gemcitabine in pancreatic adenocarcinoma cell lines, namely, BxPC-3, PANC-1, and MIA PaCa-2, and examined the outcome measures for proliferation and apoptosis as well as equilibrative nucleoside transporter 1 (ENT1) protein for evaluating the facilitation of Gemcitabine uptake by the cancer cells.

## 2. Materials and Methods

### 2.1. Cell Lines and Treatments 

BxPC-3 (CRL-1687™), PANC-1 (CRL-1469™), and MIA PaCa-2 (CRL-1420™) cell lines were obtained from ATCC (Manassas, VA, USA). BxPC-3 was maintained in RPMI-1640 (Invitrogen, Carlsbad, CA, USA) and both PANC-1 and MIA PaCa-2 were maintained in DMEM (Invitrogen, Carlsbad, CA, USA) media supplemented with 10% FBS and 1% penicillin-streptomycin solution. All the cell lines were grown in the presence of 5% CO_2_ at 37 °C. For the 2D growth experiment, all the pancreatic cell lines (10,000 cells/well) were plated in a 96-well format; 24 h later, the cells were treated with selenium in the form of methylseleninic acid (MSeA; Sigma, St. Louis, MO, USA), at a dose range of 2.5 µM–20 µM, and Gemcitabine hydrochloride (Sigma, St. Louis, MO, USA), at a dose range 0.01 µM to 10 µM, alone and in combinations for 24 h in triplicate. The three cell lines were monitored for growth inhibition at 24, 48, and 72 h time points following treatments. For 3D growth, BxPC-3 cells were plated in BD Matrigel^TM^ matrix (BD Biosciences, Bedford, MA, USA) and incubated for 30 min prior to the addition of the growth medium. These cells were treated with MSeA and Gemcitabine for 24 h, as mentioned above. The dose ranges were optimized to ensure they spanned below and above the EC_50_ of each drug (Figures S1 and S2).

### 2.2. Cell Growth Inhibition (MTT Assay)

Following treatments, 3-(4,5-dimethythiazol-2-yl)-2,5-diphenyl tetrazolium bromide (MTT; Sigma, St. Louis, MO, USA) (50 μg/well) was added to cell cultures containing phenol red-free plain RPMI-1640 medium under controlled lighting and incubated for 3 h at 37 °C in the dark. DMSO solution was added to dissolve the insoluble formazan crystals, and absorbance was read at 570 nm with a correction at 630 nm. The assay was performed in triplicate for both 2D and 3D growths. 

### 2.3. Calculating Synergy 

Drug combination synergy was quantified using two different reference models: MuSyC [32,33] and HSA [34]. Calculations were performed using the synergy Python package (v0.5.1) [35]. The MuSyC model was fit using physiologically relevant and manually determined bounds for some parameters: h bounds = (0.0001, 10,000), α bounds = (0.0001, 10,000), and γ bounds = (0.001, 1000). MuSyC synergy parameters were interpreted using standard thresholds. For synergistic potency, 0 < alpha < 1 indicates antagonism, while alpha > 1 indicates synergy. For synergistic efficacy, beta < 0 indicates antagonism, while beta > 0 indicates synergy. Synergistic efficacy is generally more important at high doses, while synergistic potency is generally more important at intermediate doses [33]. HSA synergy was calculated using the raw combination response data, compared to the mean monotherapy response value for each drug. Unlike MuSyC, which quantifies synergy using dose-independent parameters, HSA results in a distinct synergy value at each dose. HSA < 0 indicates antagonism, while HSA > 0 indicates synergy. All data and code used to generate synergy results and figures are in File S1.

### 2.4. Statistical Analysis for Synergy 

The 95% confidence intervals for MuSyC synergy parameters were obtained by Monte Carlo resampling by setting bootstrap iterations = 500. The lower bound of each parameter corresponded to the 2.5th percentile, while the upper bound corresponded to the 97.5th percentile. The same MuSyC bootstrap resamplings were also used to obtain single-drug dose-response confidence intervals. For these, the lower bound dose-response corresponded to the 2.5th percentile response at each measured dose, and the upper bound corresponded to the 97.5th percentile. The *p*-values for HSA were calculated using a one-sample, two-tailed Student’s *t*-test using the triplicate HSA values at each dose. The null hypothesis is that the mean HSA equals zero, while the alternative is that the mean HSA is not zero.

### 2.5. Immunoblotting

Control and treated BxPC-3 cells were harvested by scraping and washing with cold PBS containing protease inhibitors. Cell lysates were prepared in RIPA lysis buffer, as described earlier [21]. Equal amounts of protein (50 μg) were separated on 10% SDS-PAGE gels and transferred to PVDF membranes. Primary antibodies against cleaved-PARP (Asp214) (Cell Signaling, Danvers, MA, USA), hENT1 (F-12) and PCNA (F-2) (Santa Cruz Biotechnology, Dallas, TX, USA), and β-actin (Proteintech, Chicago, IL, USA) were reacted at 1:1000 with blots. The HRP-conjugated, anti-rabbit or anti-mouse secondary antibodies (Cell Signaling, Danvers, MA, USA) were used at a dilution of 1:3000. Band expressions were developed using Pierce ECL reagents (Thermo Scientific, Rockford, IL, USA). 

## 3. Results

**Figure 1:**
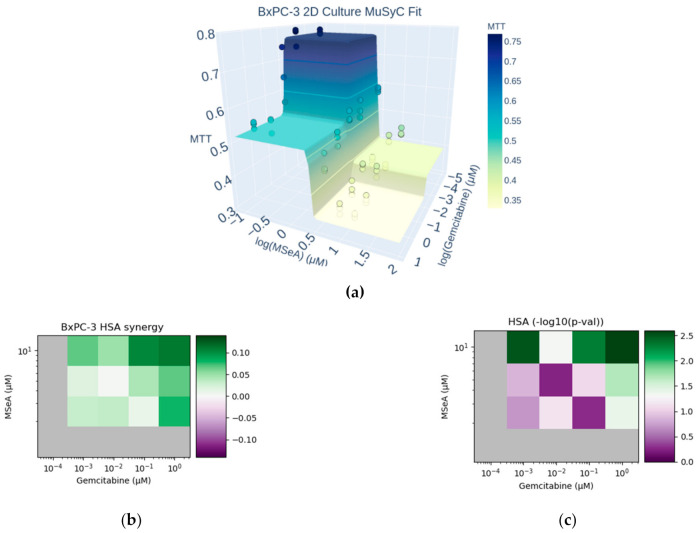
MuSyC and HSA synergy of Gemcitabine + MSeA in BxPC-3 cells grown in 2D culture. (**a**) Scatter points show raw response data, while the surface corresponds to the best MuSyC fit following 24 h treatments. Dose axes are on a log scale. A fully interactive version of this figure is in File S2. The MuSyC parameters used to generate this surface are given in Table S1. Synergistic efficacy can be seen by the fact that the deepest response is seen at high concentrations of the combination of Gemcitabine + MSeA. (**b**) Dose-dependent HSA synergy, averaged over triplicate measurements. The gray values correspond to treatment with a single drug, where synergy is not definable. Values with HSA > 0 are synergistic, while those with HSA < 0 are antagonistic. Raw HSA values are reported in Table S2. (**c**) Statistical significance of the HSA synergy from panel (**b**), determined by two-sided, one-sample t-test. Color corresponds to -log10(p). The color scale is centered so that white corresponds to *p* = 0.05, green corresponds to *p* < 0.05, and purple corresponds to *p* > 0.05.

We assessed the synergy of Gemcitabine + MSeA in BxPC-3 cells grown in 2D culture using MuSyC and HSA (Figure 1). An interactive 3D plot of the combination dose response and MuSyC fit is available in File S2. By MuSyC, the combination had synergistic efficacy β = 0.17 (95% CI: 0.10–0.24, Table S1). This indicates that at doses higher than the EC_50_ of each drug (approximately 0.1 μM of Gemcitabine and 2.5 μM of MSeA, Table S1), the combination can achieve 17% greater efficacy than the stronger drug (MSeA) alone. However, also by MuSyC, the combination showed antagonistic potency. The presence of Gemcitabine slightly decreased the potency of MSeA (α12 = 0.95, 95% CI: 0.88–0.98, Table S2). Conversely, the effect of MSeA on Gemcitabine’s potency was not statistically identifiable as synergistic or antagonistic (α21 had a 95% CI: 0.28–1.22, Table S3). All MuSyC parameter fits and their best values are given in Table S1.

Concomitant with the combination’s synergistic efficacy, HSA rated the combination as synergistic at high doses of each drug (Figure 1b,c). At low and intermediate doses, the combination was not significantly synergistic or antagonistic by HSA, indicating that the slight potency antagonism detected by MuSyC was not strong enough to be registered by the HSA model. The largest and most significant improvement of the combination over monotherapy was observed at the highest tested concentration: 10 μM of MSeA plus 1 μM of Gemcitabine.

We additionally tested synergy of these drugs in BxPC-3 cells at 24, 48, and 72 h, as well as in MIA PaCa2 and PANC-1 cell lines at 24 h (Figures S1 and S2). The results at all the timepoints for BxPC-3 showed synergy by HSA (Figure S1, Table S4) at almost all tested doses. MuSyC synergy was less conclusive, though it indicated that the MSeA improved the potency of Gemcitabine at 24 h, while Gemcitabine improved the potency of MSeA at 48 h in BxPC-3 (Table S3). In both MIA PaCa2 and PANC-1 cells, Gemcitabine had little effect (Figure S2). Further, the combination in these cell lines showed no statistically detectable synergy by MuSyC or HSA (Figure S2, Table S1). Best fits and 95% confidence intervals of each drug alone, in each cell line and time point, are shown in Figures S3 and S4. Interactive 3D visualizations of the MuSyC fits for each combination are given in Files S3–S7.

Next, we decided to examine the impact of combinations on growth in 3D. We tested whether or not this synergism is also reflected in 3D growth conditions. Since MSeA’s effects alone and in combination were promising in the BxPC-3 cell line, we limited our 3D experiments to this cell line only. Unlike the 2D growth, MuSyC was unable to statistically distinguish either synergistic or antagonistic efficacy or potency (Table S1). We speculated this was because of the uncertainty in both E1 (the maximum effect of Gemcitabine) and E3 (the maximum effect of a combination of high doses of both drugs).

**Figure 2:**
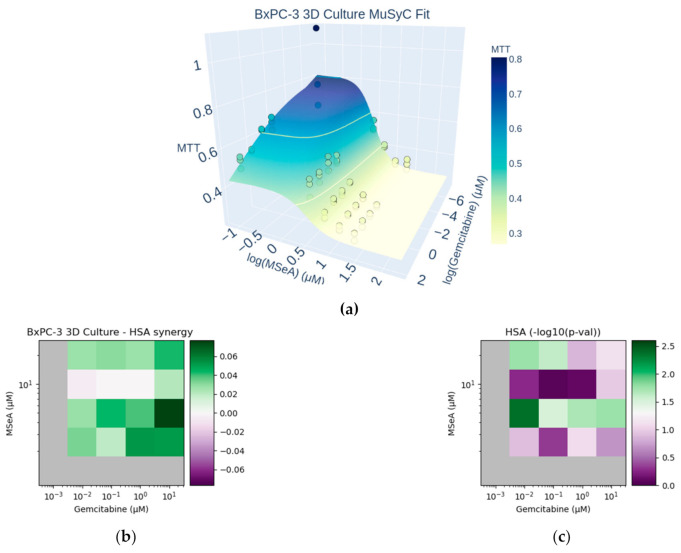
MuSyC and HSA synergy of Gemcitabine + MSeA in BxPC-3 cells grown in 3D culture. (**a**) Scatter points show raw response data, while the surface corresponds to the best MuSyC fit following 24 h treatments. A fully interactive version of this figure is in File S8. Best fits and 95% confidence intervals of gemcitabine and MSeA alone in 3D culture are shown in Figure S5. The MuSyC parameters used to generate this surface are given in Table S5. (**b**) Dose-dependent HSA synergy, averaged over triplicate measurements. Raw HSA values are reported in Table S6. (**c**) Statistical significance of the HSA synergy from panel (**b**), determined by two-sided, one-sample *t*-test.

By HSA, however, the combination was deemed synergistic at almost all tested doses (Figure 2b). This synergy was significant at 5 μM of MSeA plus all tested concentrations of Gemcitabine, and at 20 μM of MSeA combined with ≤0.1 μM Gemcitabine. 

**Figure 3:**
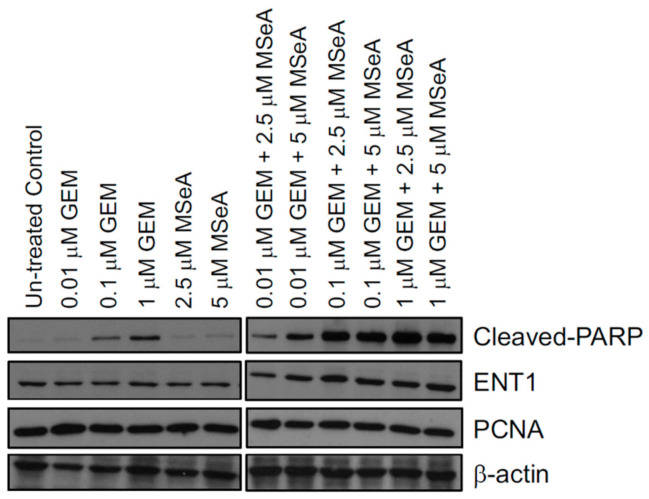
Effect of MSeA and Gemcitabine (GEM) individually and in combination on markers for apoptosis, proliferation, and ENT1 in BxPC-3 cells following 24 h treatment (n = 1). Combinations showed an increase in cleaved PARP protein expression as well as ENT1 protein expression and a moderate decrease in PCNA.

Furthermore, we evaluated the impact of selenium alone and in combination with Gemcitabine in BxPC-3 cells on relevant biological markers such as cleaved-poly ADP ribose polymerase (PARP) for apoptosis and proliferating cell nuclear antigen (PCNA) for proliferation as well as equilibrative nucleoside transporter 1 (ENT1) protein for examining the facilitation of Gemcitabine uptake by the cancer cells. Gemcitabine and MSeA alone showed a dose-dependent increase in cleaved PARP levels; however, the band intensities were lower for the latter treatment. Combinations of MSeA and Gemcitabine revealed a much stronger impact on cleaved PARP (Figure 3). Similarly, for ENT1, the combination of MSeA and Gemcitabine showed a higher level compared to monotherapy. On the other hand, PCNA was moderately decreased by combinations compared to each monotherapy. These markers were, however, not changing in a dose-dependent manner (Figure 3).

## 4. Discussion

Drug combinations are an emerging cornerstone in cancer therapy due to the ability of drugs to synergistically or additively target distinct pathways or subpopulations of patients [36]. Since Gemcitabine is a standard first-line chemotherapy for pancreatic cancers, finding drugs that synergize with it may lead to improved overall survival. One of the first randomized phase-3 trials used Erlotinib plus Gemcitabine to demonstrate statistically significantly improved survival in advanced pancreatic cancer by adding any agent to Gemcitabine [6]. Followed by another phase-3 trial in patients with metastatic pancreatic adenocarcinoma, Gemcitabine plus nab-Paclitaxel significantly improved overall survival, progression-free survival, and response rate compared to Gemcitabine alone, but rates of peripheral neuropathy and myelosuppression were increased [3]. However, another clinical study comparing Gemcitabine and Gemcitabine plus Capecitabine was recommended as the standard first-line option in locally advanced and metastatic pancreatic cancer but had a side effect of hand–foot syndrome in some patients [4,5]. Additionally, a comparison of randomized, controlled trials of four Gemcitabine-based combinations, Gemcitabine plus a platinum compound or 5-fluorouracil or irinotecan or capecitabine, revealed that no particular combination was significantly superior to another, but the indirect evidence suggests some important trends. The strongest trends on indirect comparison were towards favoring gemcitabine plus capecitabine or Gemcitabine plus a platinum compound over Gemcitabine plus irinotecan, and to a lesser degree, over gemcitabine plus 5-fluorouracil [37]. Moreover, the effectiveness of Gemcitabine may also be improved by combining it with evidence-based complementary agents [38]. Some of the proposed combining agents include resveratrol, epigallocatechin gallate, vitamin B9, capsaicin, quercetin, sulforaphane artemisinin, garcinol, thymoquinone, and emodin as well as aspirin or metformin combined with Gemcitabine [38]. More validation is, however, required for these combinations prior to approval for clinical studies.

Based on the efficacy of selenium compounds against several cancer types, we sought to evaluate the synergy between MSeA, a monomethyl form of selenium, and Gemcitabine on growth of known pancreatic adenocarcinoma cell lines. As demonstrated here, MSeA was effective on its own in BxPC-3 cells but its combination with Gemcitabine achieved stronger results than either drug tested alone in BxPC-3 cells growing in both 2D and 3D cell cultures. Conversely, PANC-1 and MIA PaCa2 cells had different outcomes. In these cell lines, Gemcitabine had virtually no effect, regardless of co-treatment with MSeA, and no synergy was detected. By contrasting the signaling responses of these cell lines in response to treatment, future work may be able to pinpoint specific mechanisms underlying synergy of this combination. In particular, we will focus on the oxidative stress-related pathway including the impact on HIF-1α and its downstream proteins, as previously demonstrated by us for MSeA [21].

Importantly, the synergistic efficacy of the combination of selenium and Gemcitabine in BxPC-3 does not come at the cost of severe antagonistic potency. In clinical settings, treatment of pancreatic cancer patients with Gemcitabine often leaves residual disease. Therefore, combinations that achieve synergistic efficacy and, thus, a greater depth of response, as demonstrated in our study, should be prioritized to improve these clinical outcomes. Our synergy data from growth measurements in BxPC-3 cells were also reflected in protein expression for proliferation as well as apoptosis when detected by PCNA and cleaved PARP protein expression, respectively. Further, moderately increased hENT1 protein expression could also have promoted the cellular uptake of Gemcitabine in BxPC-3 cells, which resulted in further enhanced MSeA + Gemcitabine-induced cell death. Most of the studies show enhanced hENT1 protein expression after 48 h treatments of drug combinations with Gemcitabine [39,40], but here we were able to see a moderate increase at the 24 h time point. 

It is imperative to conduct more than one assessment to support the efficacy of combinations of drugs; in this study, we were able to demonstrate that via MuSyC Synergy and HSA methods (measuring mitochondrial activity and viability via MTT assay) as well as by evaluating cleaved PARP levels (measuring apoptosis) following MSeA and Gemcitabine treatments in BxPC-3 cells. Future studies will focus on investigating changes in mitochondrial potential and activation of specific caspase(s). Other studies [28,29] have reported the impact of combinations of selenium and Gemcitabine; yet, the doses chosen were higher and took longer to show an impact in the pancreatic cancer cell lines. This may, in part, be attributed to the properties of the selenium form used in these studies. Most of the experiments testing potential drug efficacy are performed in cells growing on plastic (2D), which may not reflect what might be expected in vivo; therefore, conducting experiments in a 3D platform offers a better assessment for screening possible drug combinations. Moreover, in our study, it was clear that both of the compounds were able to reach the cells grown in the 3D matrix, and the combinations outperformed the monotherapy. This positively improves the chances of obtaining better outcomes of the same combinations in future in vivo study designs using pancreatic cancer models. The in vivo studies are crucial for translating outcomes to human clinical trials. 

In addition, further studies elucidating the distinct mechanisms underlying selenium and Gemcitabine combination synergy in 3D platform can strengthen the improvement in the proposed combination’s response. In particular, we will focus on investigating oxidative stress response and caspase activation in MSeA and Gemcitabine combination therapy.

## 5. Conclusions

Combining MSeA with Gemcitabine offers better synergy efficacy than each drug examined alone in BxPC-3 cells. The methodology on Synergy described for MSeA and Gemcitabine corroborates, in part, signs of proliferation, apoptosis, and ENT1 expression in BxPC-3 cells. The combination was consistently synergistic in BxPC-3 cells, at different timepoints and in both 2D and 3D cell cultures. Conversely the combination showed no synergy in other pancreatic cancer cell lines. Collectively, these results likely point to unique molecular characteristics of BxPC-3 cells, and further studies are needed to address this point.

## Data Availability

All data and code are available in File S1.

## Supplementary Materials

The following are available online at https://www.mdpi.com/article/10.3390/biomedicines10010149/s1. Figure S1: Synergy in BxPC-3 cells at 24, 48, and 72 h. Figure S2: Synergy in MIA PaCa2 and PANC-1 cell lines. Figure S3: Monotherapy response in 2D culture. Figure S4: Monotherapy response at 24, 48, and 72 h. Figure S5: Monotherapy response in 3D culture. File S1: Source code and data. File S2: BxPC-3 2D culture MuSyC Dose Response. File S3: MIA PaCa2 MuSyC Dose Response. File S4: PANC-1 MuSyC Dose Response. File S5: BxPC-3 24-h MuSyC Dose Response. File S6: BxPC-3 48-h MuSyC Dose Response. File S7: BxPC-3 72-h MuSyC Dose Response. File S8: BxPC-3 3D culture MuSyC Dose Response. Table S1: MuSyC fits. Table S2: HSA synergy. Table S3: Timeseries MuSyC. Table S4: Timeseries HSA. Table S5: 3D Culture MuSyC. Table S6: 3D Culture HSA.
