# Supplementary material for: Selenium Induces Pancreatic Cancer Cell Death Alone and in Combination with Gemcitabine
Source: Biomedicines. 2022 Jan 11;10(1):149. doi: 10.3390/biomedicines10010149 (PMC8773897; doi:10.3390/biomedicines10010149)
Supplement: Supplementary file 1 [file biomedicines-10-00149-s001.zip › Supplemental_Captions.pdf]

Supplementary Materials Captions

Figure S1: Synergy in BxPC-3 cells at 24, 48, and 72 hours. (**a**) MuSyC fit of BxPC-3 cells treated with a combination of gemcitabine + MSeA for 24 hours. (**b**) HSA synergy of the combination from (a). (**c**) Statistical significance of the HSA synergy from panel (b). (**d**)-(**f**) Synergy results fit of the same combination as (a) after 48 hours. (**g**)-(**i**) Synergy results for the same combination as (a) after 72 hours.

Figure S2: Synergy in MiaPaca2 and Panc1 cell lines. (**a**)-(**c**) Synergy results (as in Figure S1) for MiaPaca2 cells in 2D culture at 24 hours. (**d**)-(**f**) Synergy results for Panc1 cells at 24 hours.

Figure S3: Monotherapy response in 2D culture. Best fit dose response curves for (**a**)-(**c**) gemcitabine and (**d**)-(**f**) MSeA alone in BxPC-3, MiaPaca2, or Panc1 cells grown in 2D culture. The solid line represents the best fit Hill equation parameters based on the MuSyC model fit to the data. The dotted lines represent the 95% confidence interval (the lower curve is the 2.5th quantile, and the upper curve is the 97.5th quantile) for the response curve fits across 500 bootstrap iterations. Raw data are plotted as circles. Table S1 lists the Hill equation parameters corresponding to the best fit, and their confidence intervals.

Figure S4: Monotherapy response at 24, 48, and 72 hours. Best fit dose response curves, as in Figure S3, for (**a**)-(**c**) gemcitabine and (**d**)-(**f**) MSeA alone in BxPC-3 at 24, 48, or 72 hours. Table S3 lists the Hill equation parameters corresponding to the best fit, and their confidence intervals.

Figure S5: Monotherapy response in 3D culture. Best fit dose response curves, as in Figure S3, for (**a**) gemcitabine and (**b**) MSeA alone in BxPC-3 cells grown in 3D culture. Table S5 lists the Hill equation parameters corresponding to the best fit, and their confidence intervals.

File S1: Source code and data. All the code and data necessary to reproduce synergy calculations and figures.

File S2: BxPC-3 2D culture MuSyC Dose Response. A fully interactive (rotation, zoom, pan) 3D plot of the dose-response of BxPC-3 cells in 2D culture, as well as the best fit MuSyC surface.

File S3: MiaPaca2 MuSyC Dose Response. A fully interactive (rotation, zoom, pan) 3D plot of the dose-response of MiaPaca2 cells in 2D culture, as well as the best fit MuSyC surface.

File S4: Panc1 MuSyC Dose Response. A fully interactive (rotation, zoom, pan) 3D plot of the dose-response of Panc1 cells in 2D culture, as well as the best fit MuSyC surface.

File S5: BxPC-3 24-hour MuSyC Dose Response. A fully interactive (rotation, zoom, pan) 3D plot of the dose-response of BxPC-3 cells treated for 24 hours, as well as the best fit MuSyC surface.

File S6: BxPC-3 48-hour MuSyC Dose Response. A fully interactive (rotation, zoom, pan) 3D plot of the dose-response of BxPC-3 cells treated for 24 hours, as well as the best fit MuSyC surface.

File S7: BxPC-3 72-hour MuSyC Dose Response. A fully interactive (rotation, zoom, pan) 3D plot of the dose-response of BxPC-3 cells treated for 24 hours, as well as the best fit MuSyC surface.

File S8: BxPC-3 3D culture MuSyC Dose Response. A fully interactive (rotation, zoom, pan) 3D plot of the dose-response of BxPC-3 cells in 3D culture, as well as the best fit MuSyC surface.

Table S1: Cell Line MuSyC. The MuSyC best fit parameters, 95% upper- and lower-bound estimates for each parameter, and fit quality metrics for BxPC-3, MiaPaca2, and Panc1 cell lines.

Table S2: Cell Line HSA. The dose-dependent HSA synergy values for BxPC-3, MiaPaca2, and Panc1 cell lines.

Table S3: Timeseries MuSyC. The MuSyC best fit parameters, 95% upper- and lower-bound estimates for each parameter, and fit quality metrics for BxPC-3 cells measured at 24, 48, and 72 hours.

Table S4: Timeseries HSA. The dose-dependent HSA synergy values for BxPC-3 cells measured at 24, 48, and 72 hours.

Table S5: 3D Culture MuSyC. The MuSyC best fit parameters, 95% upper- and lower-bound estimates for each parameter, and fit quality metrics for BxPC-3 cells in 3D culture.

Table S6: 3D Culture HSA. The dose-dependent HSA synergy values for BxPC-3 cells in 3D culture.
