# Supplementary figures and images for: Selenium Induces Pancreatic Cancer Cell Death Alone and in Combination with Gemcitabine
Source: Biomedicines. 2022 Jan 11;10(1):149. doi: 10.3390/biomedicines10010149 (PMC8773897; doi:10.3390/biomedicines10010149)

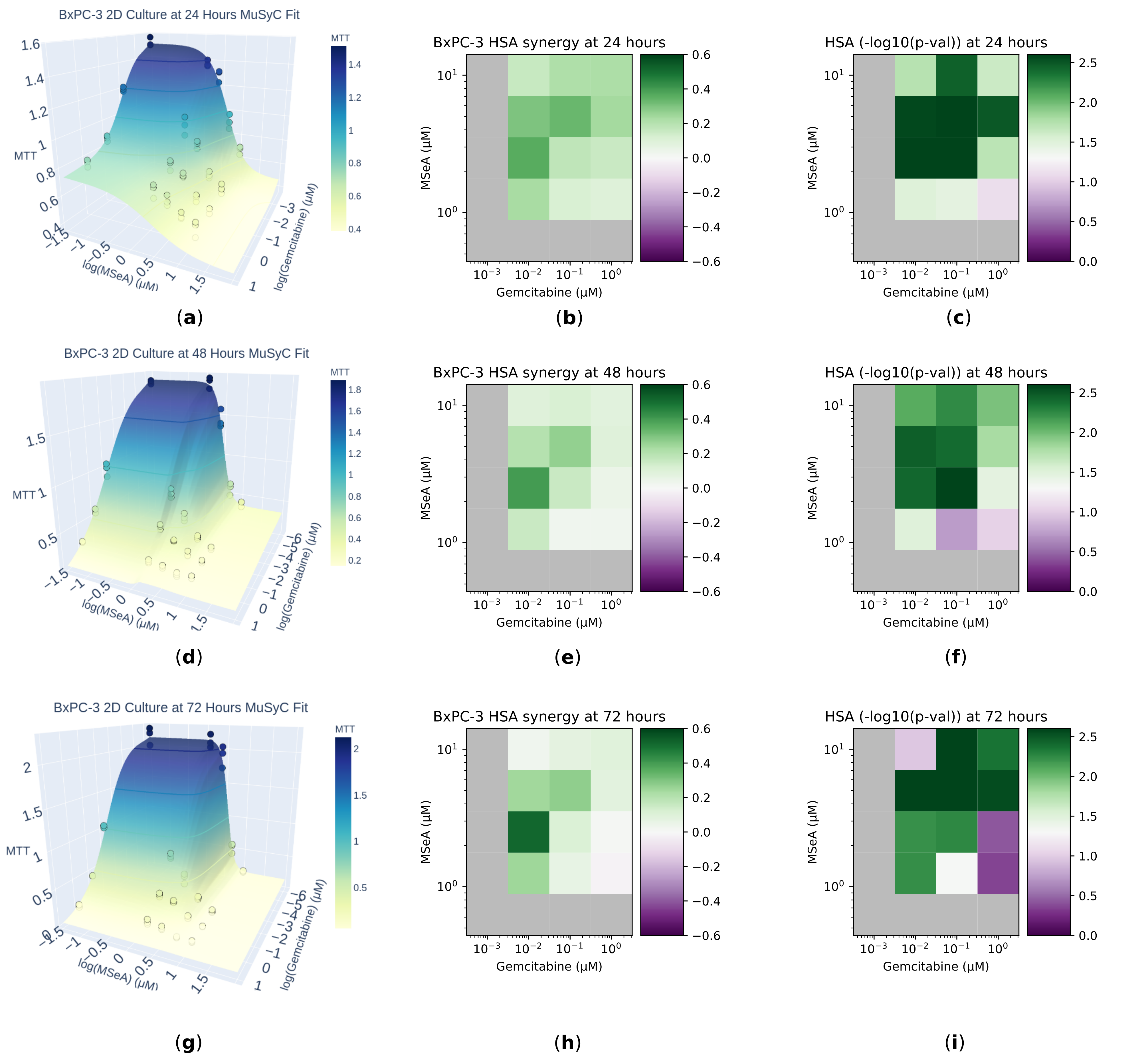

Supplement: Supplementary file 1 [file biomedicines-10-00149-s001.zip › FigureS1.png]

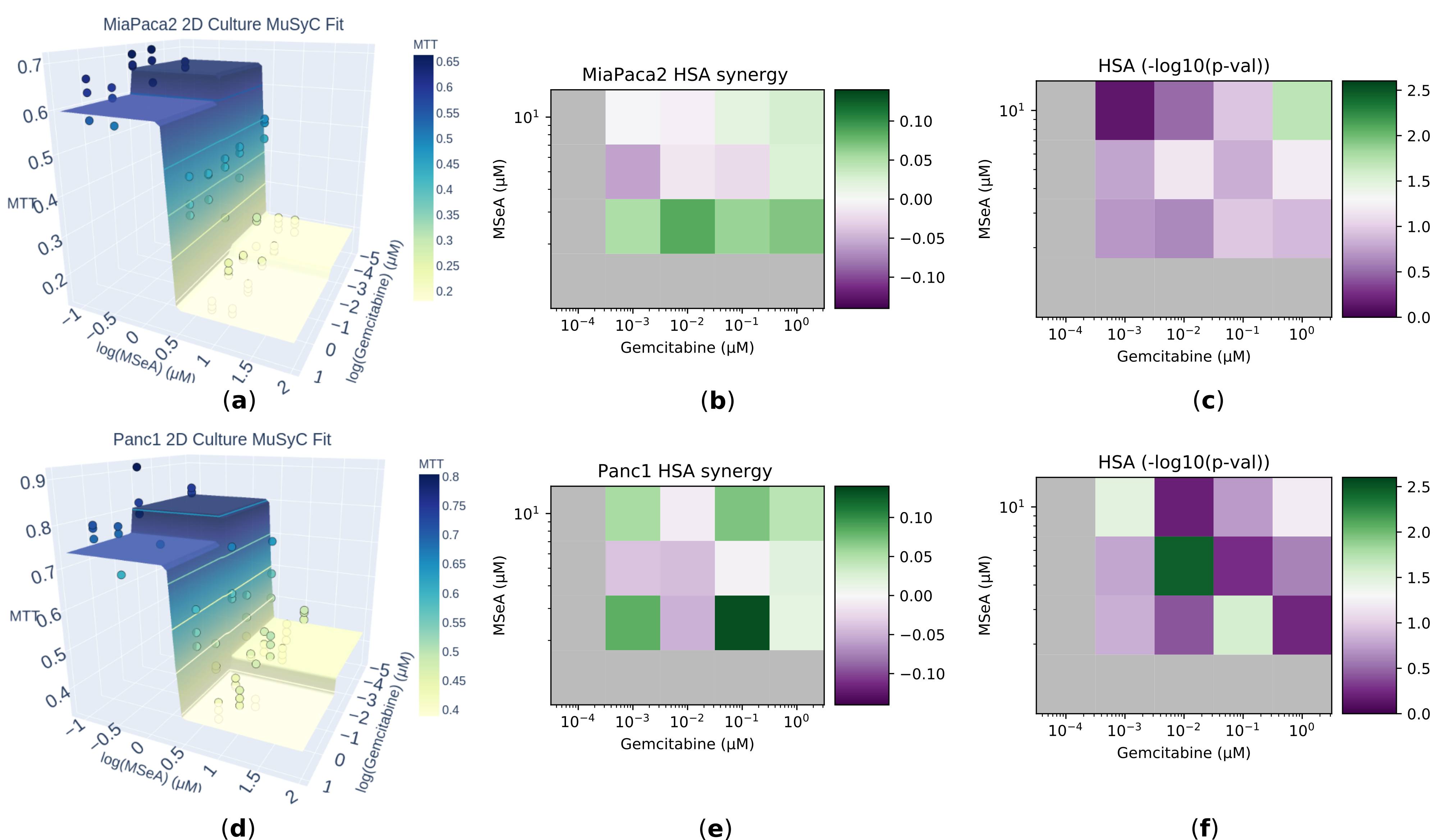

Supplement: Supplementary file 1 [file biomedicines-10-00149-s001.zip › FigureS2.png]

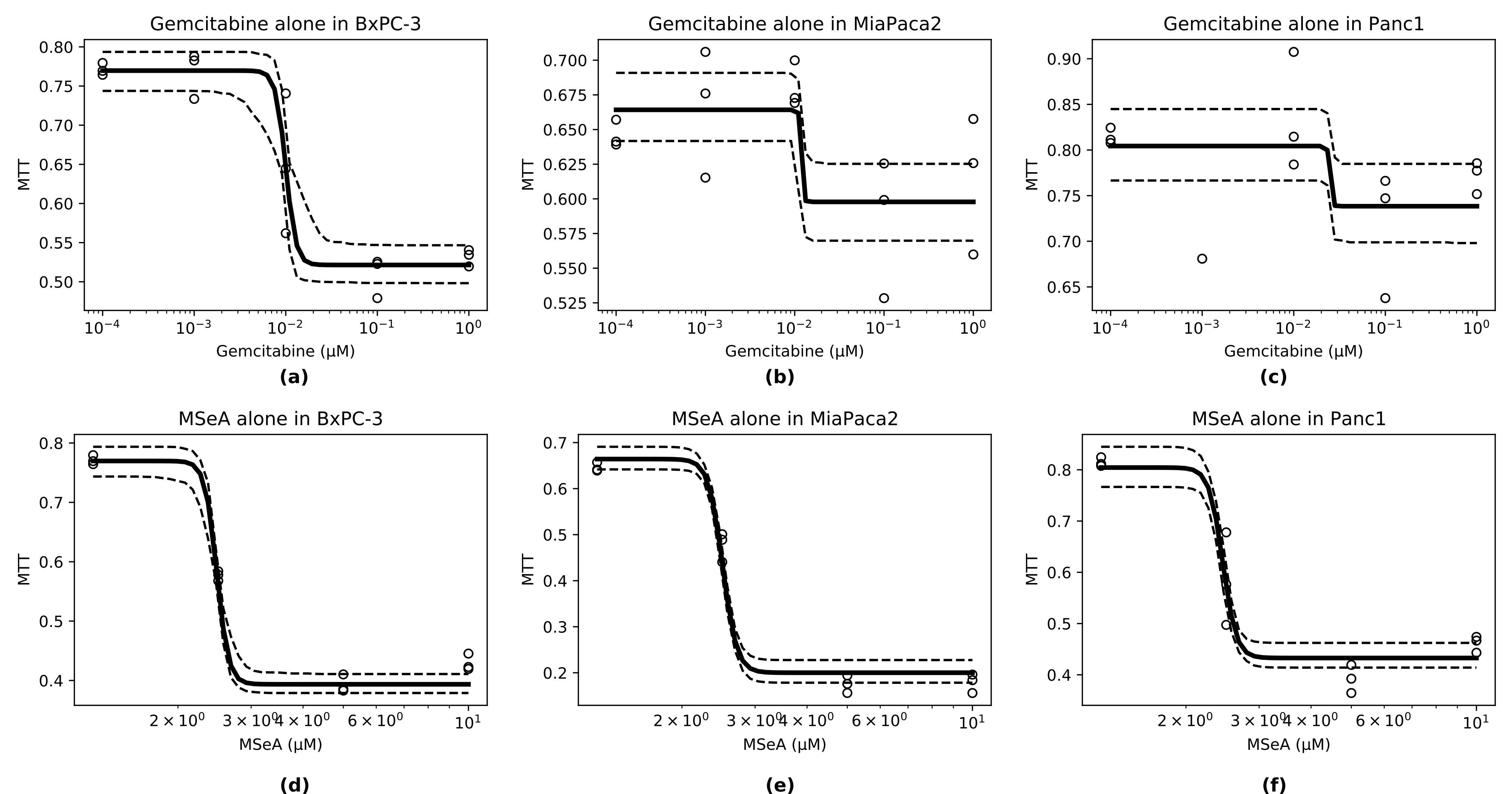

Supplement: Supplementary file 1 [file biomedicines-10-00149-s001.zip › FigureS3.png]

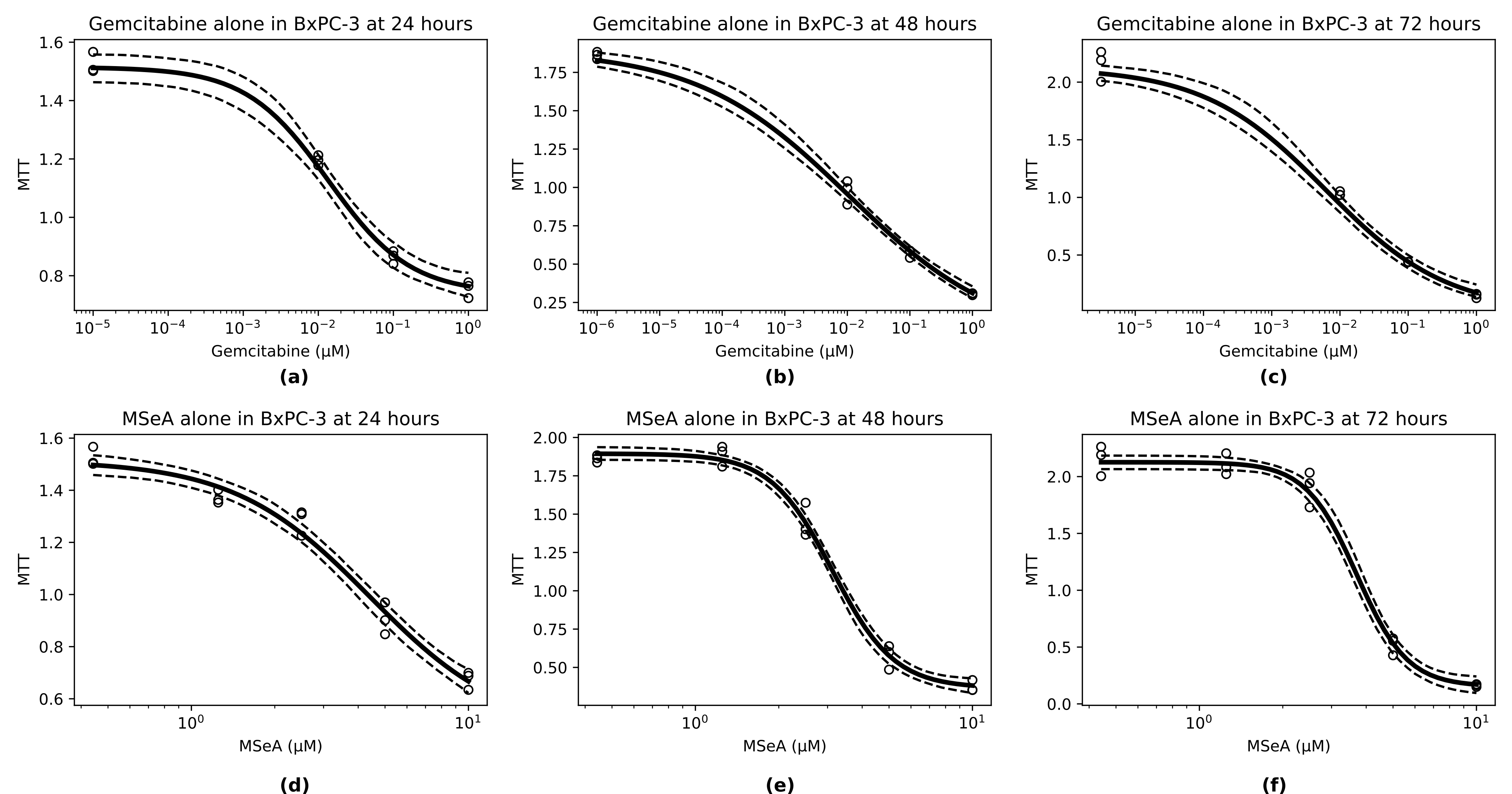

Supplement: Supplementary file 1 [file biomedicines-10-00149-s001.zip › FIgureS4.png]

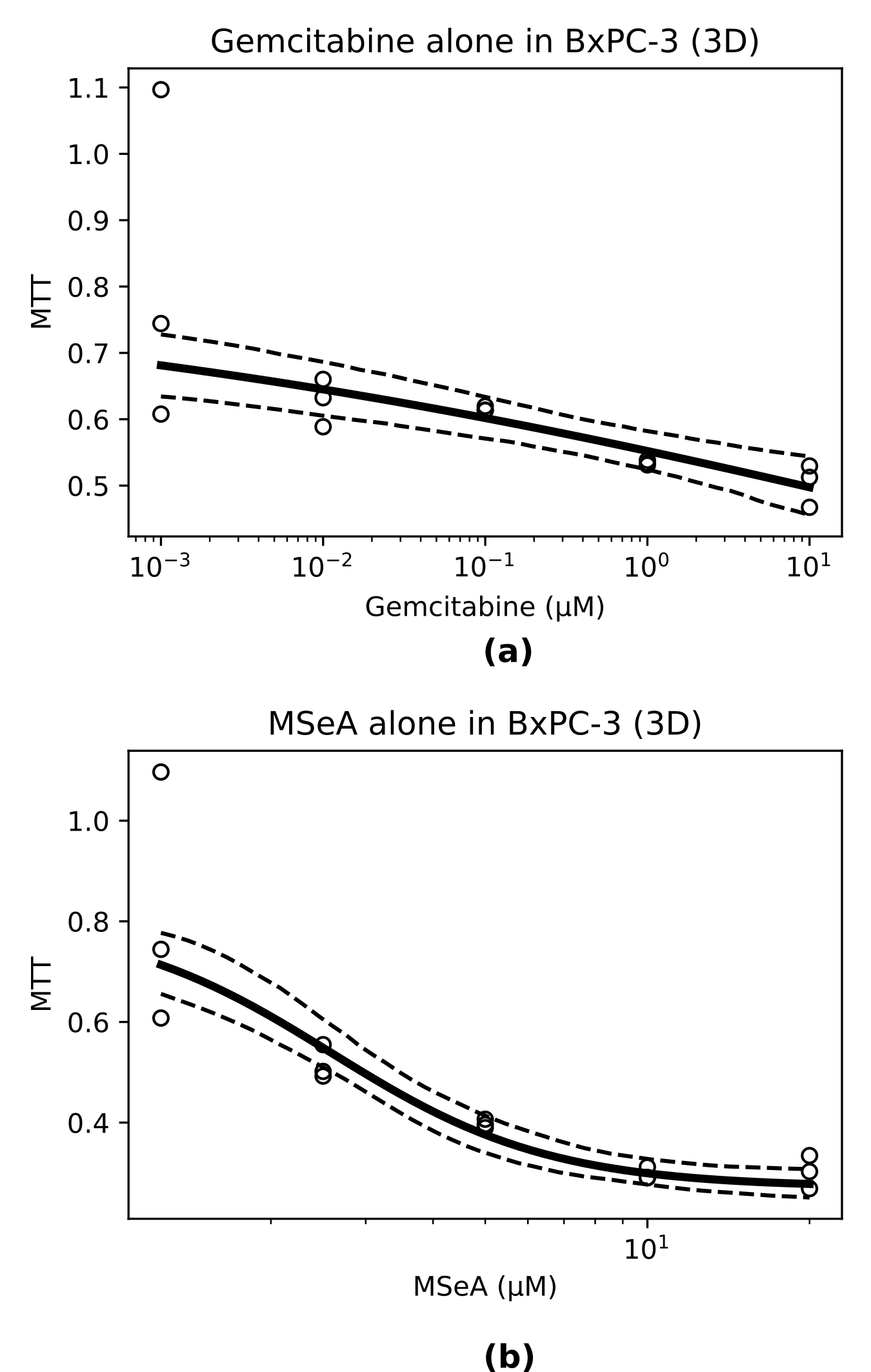

Supplement: Supplementary file 1 [file biomedicines-10-00149-s001.zip › FIgureS5.png]
